# Dietary Omega-3 Deficiency from Gestation Increases Spinal Cord Vulnerability to Traumatic Brain Injury-Induced Damage

**DOI:** 10.1371/journal.pone.0052998

**Published:** 2012-12-28

**Authors:** Zhe Ying, Cameron Feng, Rahul Agrawal, Yumei Zhuang, Fernando Gomez-Pinilla

**Affiliations:** 1 Department of Integrative Biology & Physiology, University of California Los Angeles, Los Angeles, California, United States of America; 2 Department of Neurosurgery, UCLA Brain Injury Research Center, Los Angeles, California, United States of America; Max Delbrueck Center for Molecular Medicine, Germany

## Abstract

Although traumatic brain injury (TBI) is often associated with gait deficits, the effects of TBI on spinal cord centers are poorly understood. We seek to determine the influence of TBI on the spinal cord and the potential of dietary omega-3 (n-3) fatty acids to counteract these effects. Male rodents exposed to diets containing adequate or deficient levels of n-3 since gestation received a moderate fluid percussion injury when becoming 14 weeks old. TBI reduced levels of molecular systems important for synaptic plasticity (BDNF, TrkB, and CREB) and plasma membrane homeostasis (4-HNE, iPLA2, syntaxin-3) in the lumbar spinal cord. These effects of TBI were more dramatic in the animals exposed to the n-3 deficient diet. Results emphasize the comprehensive action of TBI across the neuroaxis, and the critical role of dietary n-3 as a means to build resistance against the effects of TBI.

## Introduction

Although deficit in motor function is a common consequence of traumatic brain injury (TBI), not much is known about the influence of brain injury on motor centers in the spinal cord. We are starting to understand that TBI reduces the expression of molecules important for synaptic plasticity in the spinal cord [1], and are thus arguing for the need of broad therapeutic strategies to influence the brain and spinal cord. Here we have studied the capacity of foods to promote increased spinal cord resilience to the type of diffuse injury caused by brain trauma, in particular, the essential omega-3 (n-3) poly-unsaturated fatty acid (PUFA) docosahexaenoic acid (DHA, 22∶6n-3), which is gaining recognition for supporting neuronal function and plasticity. Inadequate consumption of dietary DHA during CNS development results in aberrations in neuronal function, and learning ability [2], [3], while dietary DHA supplementation in the adult brain aids recovery after brain injury [4], [5]. In the present study, we seek to determine whether dietary supplementation of DHA could influence the capacity of the spinal cord to cope with the effects of injury to the brain.

We used fluid-percussion injury (FPI) as an animal model of TBI since this injury promotes circuit dysfunction without extensive neuronal death [6]. Specifically, FPI results in significant reductions of brain-derived neurotrophic factor (BDNF) and its downstream effectors. BDNF is important for many aspects of neuronal function and plasticity, influencing adult neurogenesis, and providing protection after neuronal injury [7]. As we know that TBI promotes oxidative damage of the plasma membrane [8], likely influencing the membrane’s phospholipid composition, such as DHA, we used the lipid peroxidation marker 4-HNE to assess the status of the plasma membrane in response to TBI and DHA interventions [8]. We have also assessed syntaxin-3 based on its role as a modulator of neuronal membrane expansion, especially during synaptic growth [9], and assessed calcium-independent phospholipase A-2 (iPLA-2) based on its influence on membrane phospholipid biosynthesis and turnover [10].

## Results

### Synaptic Proteins (Fig. 1)

**Figure 1:**
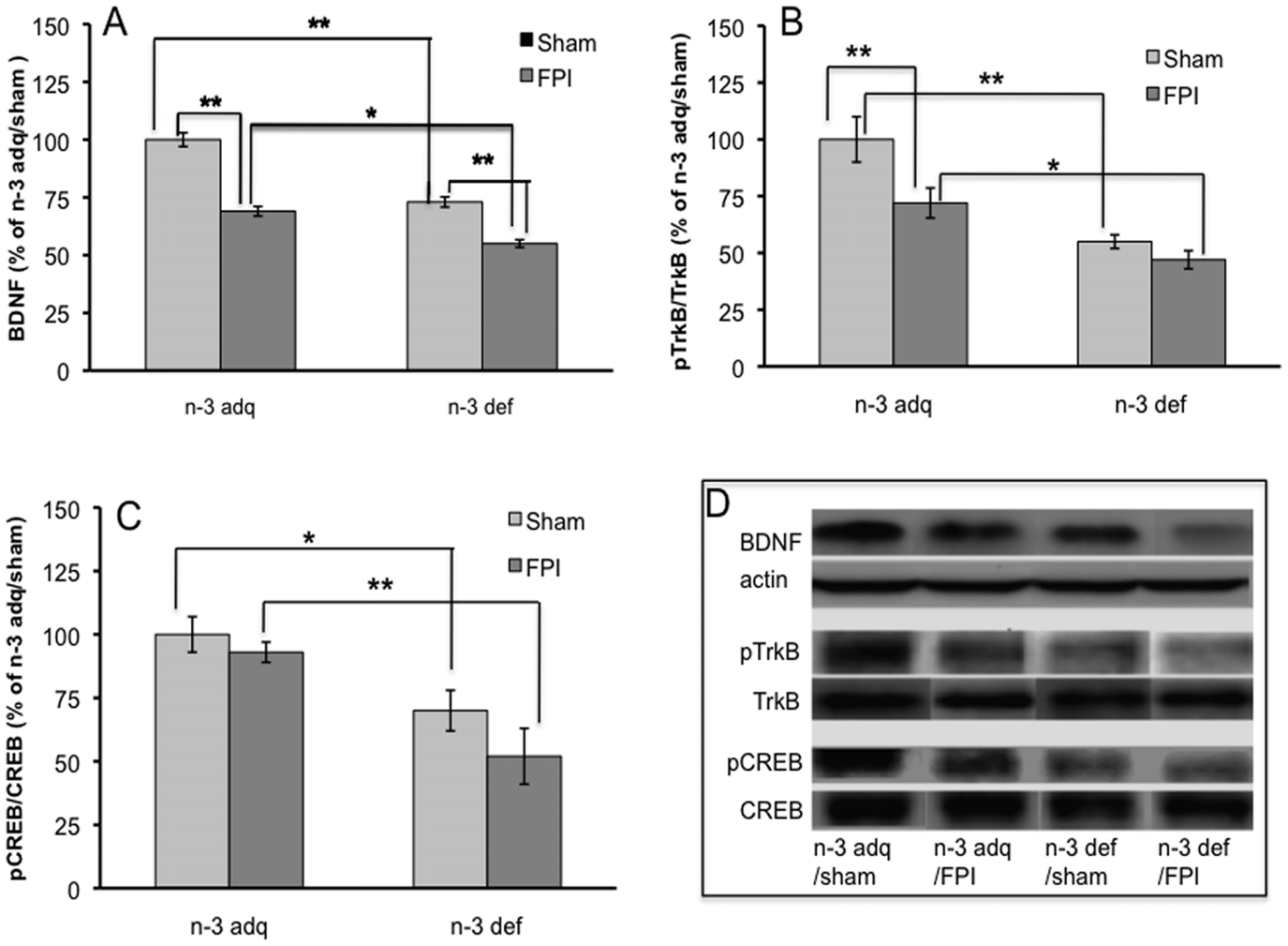
Synaptic plasticity markers BDNF (A), pTrkB (B), and pCREB (C) protein levels were assessed in the lumbar spinal cord of rats exposed to FPI, using western blot assay. (D). Representative western blot bands from experimental groups. Results were expressed as mean ± standard error of the mean (SEM), *P<0.05, **P<0.01.FPI, fluid percussion injury; n-3 def, omega 3 fatty acids deficient; n-3 adq, omega 3 fatty acid adequate. n-3 def/sham: n = 5; n-3 adq/sham: n = 6; n-3 def/FPI: n = 5; n-3 adq/FPI: n = 7.

BDNF levels were significantly reduced in the animals fed the n-3 deficient diet (n-3 def/sham, 74%, p<0.01, n = 5) as compared to the animals fed adequate n-3 (n-3 adq/sham, n = 6) (Fig. 1A). FPI reduced levels of BDNF in the n-3 deficient group (n-3 def/FPI, p<0.01, n = 5). Although FPI reduced BDNF levels in the n-3 adq rats (p<0.02, n = 7), these levels were still higher than the n-3 def group (p<0.05, Fig. 1A). The n-3 deficient diet reduced the levels of pTrkB/TrkB when compared to the n-3 adq/sham group (p<0.01, Fig. 1B). Although FPI reduced levels of pTrkB/TrkB in the group receiving adequate levels of n-3, pTrkB/TrkB levels were still higher than the n-3 def group (n-3 adq/FPI vs. n-3 def/FPI, p<0.05, Fig. 1B). Levels of pCREB/CREB were reduced in the animals exposed to the n-3 deficient diet (n-3 def/sham vs. n-3 adq/sham, p<0.05, Fig. 1C) and FPI had a tendency to reduce pCREB/CREB levels even further (Fig. 1C). Fig. 1D showed the representative western bands corresponding to protein markers and animal groups.

### Membrane Homeostasis (Fig. 2)

**Figure 2:**
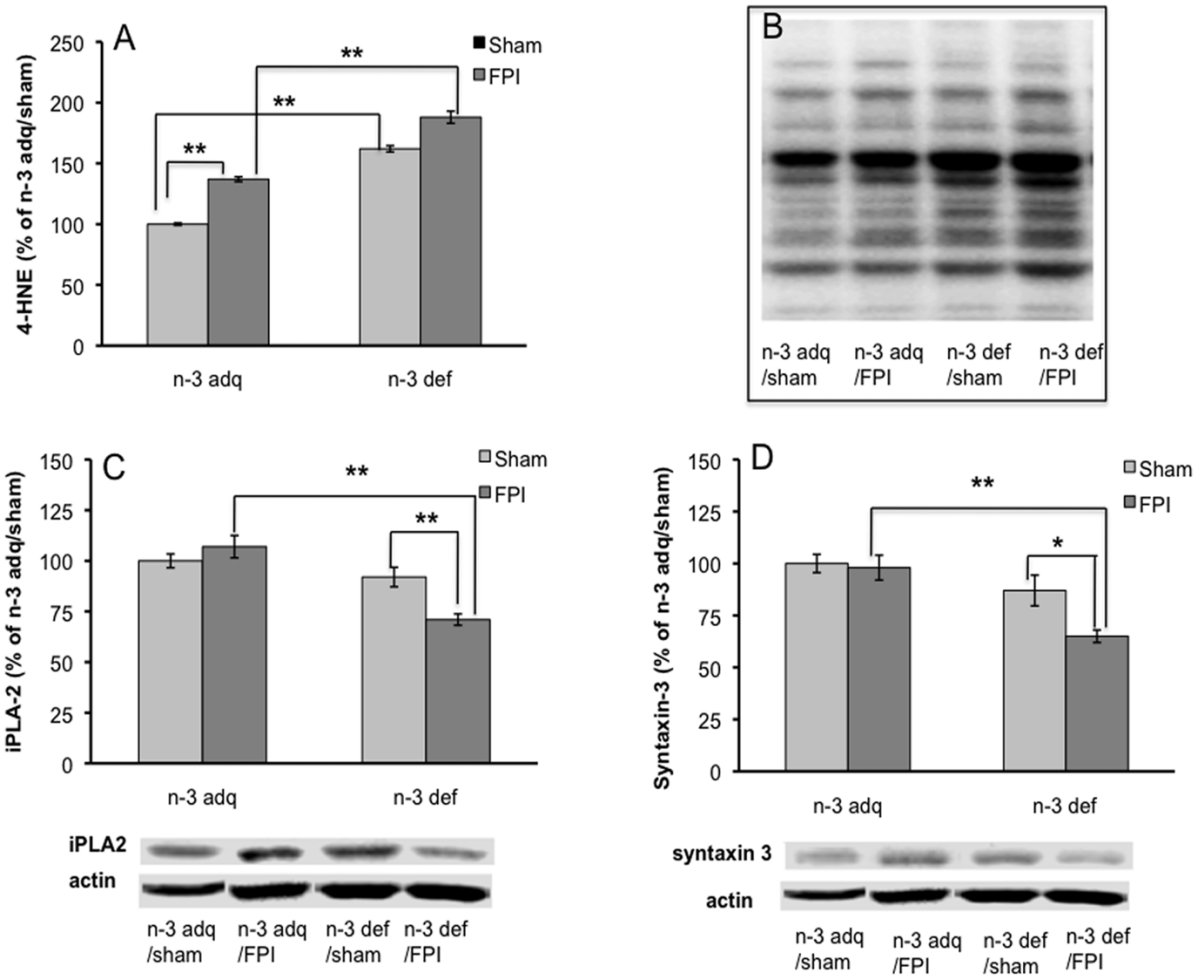
Levels of molecules related to plasma membrane homeostasis 4-HNE (A, B), iPLA2 (C), and syntaxin 3 (D) in the lumbar spinal cord of rats exposed to FPI. Results were expressed as mean ± standard error of the mean (SEM), *P<0.05, **P<0.01. FPI, fluid percussion injury; n-3 def, omega 3 fatty acids deficient; n-3 adq, omega 3 fatty acids adequate. n-3 def/sham: n = 5; n-3 adq/sham: n = 6; n-3 def/FPI: n = 5; n-3 adq/FPI: n = 7.

We assessed levels of 4-HNE which is a suitable marker of plasma membrane lipid peroxidation. Results showed that the n-3 def diet increased levels of 4-HNE in the spinal cord as compared to n-3 adq diet (p<0.01, Fig. 2A, 2B). FPI elevated 4-HNE levels even further in the n-3 def animals (p<0.01, Fig. 2A, 2B). Although FPI also elevated levels of 4-HNE in the n-3 adq group, 4-HNE levels were lower than in the n-3 def group (p<0.01, Fig. 2A, 2B).

We measured iPLA2 levels based on its involvement in the metabolism of membrane phospholipids [11] FPI significantly reduced the levels of iPLA2 in the animals fed n-3 deficient diet (n-3 def/FPI vs. n-3 def/sham, p<0.01). FPI had no effects on levels of iPLA-2 in the n-3 adq group suggesting a counteractive effect (Fig. 2C) such that levels of iPLA2 in the n-3 def rats exposed to FPI rats were significantly lower than their counterpart in the n-3 adq group (p<0.01, Fig. 2C).

Although the exposure to the n-3 deficient diet did not affect levels of syntaxin-3 in the sham rats relative to the adq group, FPI strongly reduced syntaxin-3 levels in the n-3 def group (n-3 def/FPI group as compared to n-3 adq/FPI group (P<0.01) and n-3 def/sham (P<0.05) groups (Fig. 2D).

### Fatty Acids in Spinal Cord (Fig. 3)

**Figure 3:**
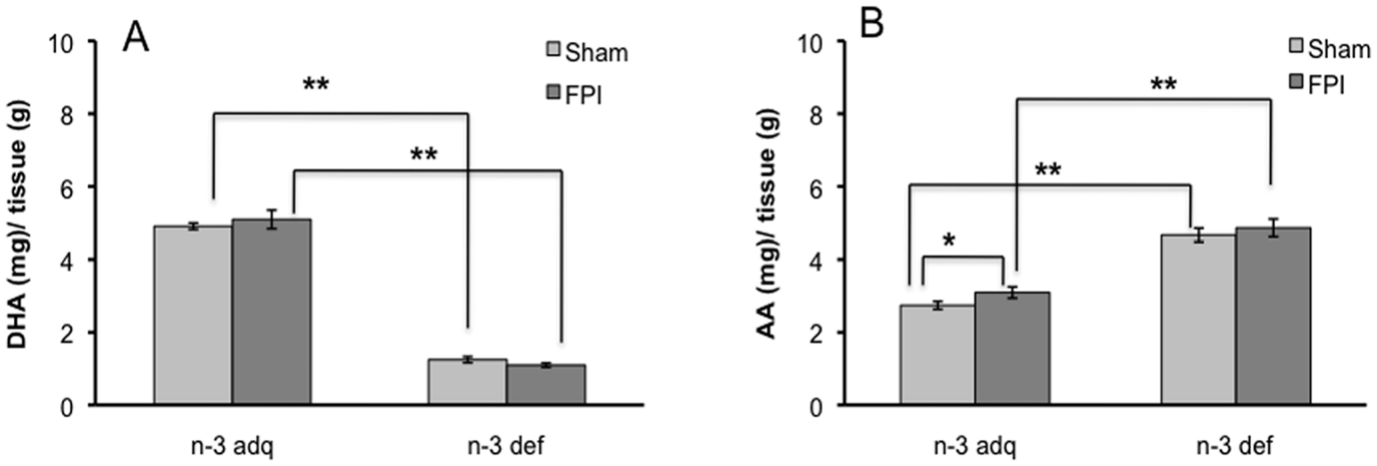
Gas chromatography was used to assess levels of DHA (A) and AA (B) in the cervical spinal cord of FPI rats. An n-3 def diet significantly decreased DHA and increased AA levels. FPI increased AA levels of n-3 adq group (p<0.05) but had no effects in n-3 def group. Data are shown as ratio of fatty acid(mg)/tissue(g). *P<0.05, **P<0.01. DHA, docosahexaenoic acid; AA, arachidonic acid. n-3 def/sham: n = 5; n-3 adq/sham: n = 6; n-3 def/FPI: n = 5; n-3 adq/FPI: n = 7.

Levels of docosahexaenoic acid (DHA, 22∶6n-3) and arachidonic acid (AA, 20∶4n-6) were measured in the spinal cord region using gas chromatography. Results showed that the levels of DHA significantly decreased in animals fed on n-3 deficient diet (n-3 def/sham). FPI did not affect levels of DHA in the n-3 def group (n-3 def/FPI) or the n-3 adq group (Fig. 3A). In turn, levels of AA were increased significantly in the sham and FPI groups exposed to the n-3 deficient diet (p<0.01, Fig. 3B). FPI also increased AA levels in the n-3 adq rats (P<0.05) (Fig. 3B).

## Discussion

We found that brain concussive injury reduces molecular substrates of plasticity in the spinal cord, and these effects were dependent on the availability of DHA in the diet. These results emphasize the comprehensive action of TBI across the neuroaxis, and the critical role of diet as a means to build resistance against the effects of TBI. According to our results, proper exposure to n-3 fatty acids during gestation and throughout maturation of the CNS is crucial for building neural resilience during adulthood. The effects of diet and TBI were observed on levels of molecules associated with the function of BDNF on synaptic plasticity, and plasma membrane homeostasis in the spinal cord.

### Synaptic Plasticity

According to our results, FPI and the diet deficient in DHA reduced protein levels of BDNF and its receptor TrkB in the SC, as well as elements related to the action of BDNF on synaptic plasticity such as syntaxin 3 and CREB, which have recognized roles in synaptic plasticity and learning and memory [12]. These results suggest that FPI reduces the capacity of the SC for plasticity. The action of the BDNF system seems crucial for mediating the action of DHA in the brain as a diet deficient in DHA has been shown to reduce activation of the BDNF TrkB receptors [3], and the capacity of the SC for learning a motorsensory task [13]. Therefore, the reduction of BDNF because of the DHA def or TBI in our study may have negative implications for the potential of the SC to functionally recover after brain or SC injury. On the other hand, the fact that DHA supplementation is related to higher levels of BDNF argues in favor of a therapeutic potential of DHA. Indeed, DHA has shown protective capacity when provided after hemisection or compression spinal cord injury by increasing the survival of neurons and improving locomotor performance [14].

### Membrane Homeostasis

DHA is a structural component of plasma membrane, and membrane bound DHA supports membrane fluidity [15], which is instrumental for neuronal signaling. The high contents of DHA and other phospholipids in the plasma membranes make the membrane a vulnerable target to lipid peroxidation. Lipid peroxidation has been linked to a disruption in membrane homeostasis and impairment of synaptic plasticity. Here, we found that FPI increased lipid peroxidation in the SC as evidenced by increased levels of 4-HNE. The phospholipase A_2_ (PLA_2_) family is involved in the metabolism of membrane phospholipids [11], and the calcium-independent PLA_2_ (iPLA_2_) plays an important role in synaptic plasticity [16], [17]. Therefore, our results showing significant changes in iPLA2 levels in the n-3 def animals undergoing FPI provide an indication for the compromise of membrane homeostasis. In turn, STX-3 is a membrane-bound synaptic protein which function is influenced by DHA [18]. The fact that the diet deficient in DHA increased lipid peroxidation and decreased syntaxin 3, suggests how a lack of membrane DHA promotes membrane instability [19]. Syntaxin 3 is positioned in the presynaptic plasma membrane to detect local changes in PUFA [18] and plays a crucial role in the docking and fusion of vesicles during synaptic transmission [20]. Therefore, our results showing that FPI and dietary n-3 affect levels of 4-HNE, iPLA2, and STX-3, suggest a potential mechanism by which TBI and diet can influence membrane homeostasis required for functional recovery after spinal cord injury.

It is important to consider that although DHA (1.2%) is the most abundant omega-3 fatty acid in our diet, there are other less abundant fatty acids as well such as eicosapentaenoic acid (EPA). EPA has also been reported to support neural repair events such as reducing axonal injury after spinal cord compression; however, its action appears less effective than DHA [21]. Given the large difference in contents of DHA (1.2%) Vs. EPA (0.24%), it is likely that the main effects of the diet are related to DHA.

The effects of the n-3 feeding can also be perceived at the levels of AA and DHA in the spinal cord, as evidenced by results showing that n-3 deficiency increased AA levels but reduced DHA levels. These results are consistent with the possibility that AA could replace DHA in the membrane. A reduction in membrane fluidity can affect transmembrane receptors such as TrkB, and this may explain why the n-3 def diet reduced TrkB activity in our study. DHA modifies the characteristics of lipid rafts by incorporating into raft domains of the membrane and influencing signaling across embedded receptors [22] such as TrkB receptors [23].

The current results emphasize the pervasive effects of brain trauma impacting CNS regions, which are distant from the lesion. These results have important implications for the design of potential treatments directed to counteract the effects of TBI. Based on results showing the comprehensive effects of brain injury in the brain and spinal cord, it appears that interventions that have the capacity to influence the entire neuroaxis can be particularly effective. As discussed above, the broad spectrum of action of the omega-3 fatty acid DHA positively influencing the brain and spinal cord appears particularly suitable for this purpose. It is critical to complement our molecular data with behavioral studies. It is known that the type of TBI used in the current study promotes deficits in cognition and gait [1] and that post-injury treatment with DHA counteracts some these deficits [8]. A period as short as 12 days of DHA following FPI has been shown to be sufficient to counteract deficits in hippocampal-dependent learning [8]. The unique aspect of our results is the demonstration that dietary DHA during CNS maturation confers resilience to neurological damage in adult life. These results have important implications to appraise the role of diet as a vulnerability factor for the outcome of TBI. It is a common observation that healing after brain or spinal cord injury is not often predictable based on the extent of the neurological damage. This implies that vulnerability factors associated with the environment and genetics have great potential to determine the outcome of CNS injured patients.

Our results show that exposure to n-3 fatty acids during gestation and throughout maturation of the CNS is important for building resilience to neurological damage incurred later on in life. Further studies are required to define whether shorter dietary DHA exposure can confer CNS protection. In conclusion, these results are important to define the broad and positive action of n-3 diet on counteracting the effects of concussive brain injury on the spinal cord.

## Materials and Methods

All experimental procedures were performed in accordance with the United States National Institutes of Health Guide for the Care and Use of Laboratory Animals and were approved by the University of California at Los Angeles (UCLA) Chancellor’s Animal Research Committee (ARC).

### Experimental Design

Two-day pregnant female Sprague-Dawley rats weighing between 280 and 300 g were obtained from Charles River Laboratories, Inc. (Portage, MI, USA). The animals were housed with 12-h light/dark cycles and a maintained temperature of 22–24°C. Animals were randomly divided into two dietary groups: omega 3 fatty acids adequate (n-3 adq) diet vs omega 3 fatty acids deficient (n-3 def) diet. The dietary treatment started with the mothers and the male offspring were weaned to the same diet as their dam. After 14 weeks, the animals were subjected to moderate FPI, and continued their diet until sacrificed one week later.

### Diet Composition

The two custom diets (n-3 def, n-3 adq) were prepared commercially (Dyets, Bethlehem, PA, USA) and contained the same basal macronutrients, vitamins, minerals, and basal fats (hydrogenated coconut and safflower oils). Vitamin-free casein Alacid 710 (NZMP North America Inc., CA, USA) was included. The n-3 adq diet contained an extra 0.5% flaxseed oil (linoleic acid), 1.2% DHA and 0.24% EPA (Nordic Naturals, Inc. Watsonville, CA, USA), relative to the n-3 def diet. (Table 1). The individual’s daily intake of DHA was about 480 mg per kilogram of animal weight.

**Table 1:** Fat composition of the experimental diets (g/100 g diet).

Fat sources	n-3 adq	n-3 def
Hydrogenated coconut oil	7.45	8.1
Safflower oil	1.77	1.9
Flaxseed oil	0.48	0
DHA	1.2	0
EPA	0.24	0
Other n-3s	0.1	0

### Fluid Percussion Injury (FPI)

FPI was performed as previously described [10]. Under deep anesthesia, A 3.0 mm diameter craniotomy was made 3.0 mm posterior to the bregma and 6.0 mm lateral (left) to the midline, and a cap was glued over the craniotomy. The cap was filled with 0.9% saline solution and a mild fluid percussion pulse (1.5 atm) was administered. The severity of injury was confirmed based on the unconscious time (less than 120 seconds) of the animal following injury before the first response to a paw inch. Sham animals underwent an identical preparation with the exception of the FPI.

### Tissue Collection

One week following FPI, rats were sacrificed by decapitation, and the cervical enlargement (C3–C6) and lumbar enlargement (L2–L6) regions were collected and stored in −70°C for lipids and protein measurements.

### Protein Analysis

BDNF, phosphorylated tyrosine kinase (pTrkB), phosphorylated cAMP response element-binding (pCREB), syntaxin-3, calcium-independent phospholipase A_2_ (iPLA-2), and 4-Hydroxynonenal (4-HNE) proteins of the right lumbar SC were analyzed using western blots as previously described [10]. Tissue was first homogenized in a lysis buffer and the total protein was separated by electrophoresis on a polyacrylamide gel and electrotransferred onto a PVDF (nitrocellulose for BDNF) membrane (Millipore, Bedford,MA). After blocking, the membranes were rinsed with TBS-T and incubated with the primary antibody for actin (Santa Cruz Biotechnology, Santa Cruz, CA, USA), BDNF (1∶300, Santa Cruz Biotechonology, Santa Cruz, CA, USA), pTrkB (1∶200, BD Biosciences, Sparks, MD, USA), TrkB (1∶500, Santa Cruz Biotechnology, Santa Cruz, CA, USA), pCREB (1∶1,000, Millipore, Bedford, MA, USA), CREB (1∶200, Millipore, Bedford, MA, USA), syntaxin-3 (1∶300, Santa Cruz Biotechnology, Santa Cruz, CA, USA), iPLA-2 (1∶200, Santa Cruz Biotechnology, Santa Cruz, CA, USA), or 4-HNE (1∶500, Santa Cruz Biotechnology, Santa Cruz, CA, USA ). Immunocomplexes were visualized by chemiluminescence using the commercial kit ECL plus (Amersham Pharmacia Biotech Inc., Piscataway, NJ, USA). Respective protein sizes were compared to the Benchmark pre-stained protein ladder (Invitrogen Technology, Carlsbad, CA, USA). Protein bands were digitally scanned and quantified using the ImageJ software. Actin was used as an internal control. The phosphorylated proteins were normalized to their respective non-phosphorylated partners.

### Fatty Acids Analysis by Gas Chromatography

The lipids content of the cervical SC were extracted and analyzed by gas chromatography. Lipids were first extracted by homogenizing tissue on ice in a 2∶1 (vol:vol) chloroform:methanol solution with 50 ug/mL of butylated hydroxytoluene added to prevent lipid oxidation. Tricosanoic acid methylester (C23∶0) was added to each sample to function as an internal standard. After extraction, lipids were methylated by heating at 90°C for 1 hour in14% (w/v) boron trifluoride-methanol reagent. The lipid contents were analyzed using the Clarus 500 gas chromatograph with a built-in Autosampler (PerkinElmer), and the total runtime for each sample was 34 min. 1uL FA methyl esters (FAME) was injected in split injection mode with a 100∶1 split ratio, and the resultant peaks were identified and quantified by comparison with the standard Supelco 37-component FAME Mix (Sigma-Aldrich, USA).

### Statistical Analysis

Protein data were expressed as a mean percentage of the control (sham/n-3 adq) group DHA and AA levels were expressed as a ratio of fatty acid (mg)/tissue (g). Significance among groups was determined using SPSS software. One-way analysis of variance (ANOVA) was performed followed by Fisher’s Least Significance Difference test. Results were expressed as mean ± standard error of the mean (SEM), n = 5–7/group. Significant difference was considered at P<0.05.
